# Novel Score-based Decision Approach in Chronic Myeloid Leukemia Patients After Acute Toxic Imatinib-induced Liver Injury

**DOI:** 10.7759/cureus.4411

**Published:** 2019-04-09

**Authors:** Nataliia Lopina, Iryna Dmytrenko, Dmytro Hamov, Dmytro Lopin, Iryna Dyagil

**Affiliations:** 1 Internal Medicine and Endocrinology, Kharkiv National Medical University, Kharkiv, UKR; 2 Hematology and Transplantology, National Research Center for Radiation Medicine of the National Academy of Medical Sciences of Ukraine, Kyiv, UKR; 3 Hematology, Cherkasy Regional Oncology Dispensary of Cherkasy Regional Council, Cherkassy, UKR; 4 Internal Medicine, SI Zaitsev V.t Institute of General and Urgent Surgery of National Academy of Medical Science of Ukraine, Kharkiv, UKR; 5 Radiation Oncohematology and Stem Cell Transplantation, National Research Center for Radiation Medicine of the National Academy of Medical Sciences of Ukraine, Kyiv, UKR

**Keywords:** acute toxic hepatitis, acute liver failure, chronic myeloid leukemia, chronic myeloid leukemia second-line therapy, tyrosine kinase inhibitors, imatinib, nilotinib, dasatinib, ponatinib, drug-induced liver disease

## Abstract

The tyrosine kinase inhibitor (TKI) imatinib in rare cases can cause acute toxic hepatitis, hepatic failure, and death. Currently, the choice of further chronic myeloid leukemia (CML) therapy in patients after acute hepatotoxicity is still a difficult question, which requires a complex individual approach based on the clinical guidelines of adverse event management. Data about the further follow-up strategy approach in patients with CML after acute toxic imatinib-induced liver injury are of concern, and at times controversial. In addition, one of the questions is about the necessity and safety of the imatinib therapy resumption after acute hepatotoxicity. In some publications, imatinib resumption without the recurrence of hepatotoxicity has been discussed; in others, imatinib resumption with the recurrence of imatinib hepatotoxicity has been mentioned. There are a few publications about the experience of administration of the second-line TKIs after acute imatinib hepatotoxicity. There are no clear data on which factors the physician’s decision should be based on in patients with CML after acute toxic imatinib-induced liver injury. Imatinib should be restarted or withdrawn, when and for whom second-line therapy should be started. The physician’s decision is usually based on the published data of similar cases, personal experience, and the severity of hepatotoxicity. We have discussed the clinical guidelines devoted to the peculiarities of the patient’s management after acute toxic imatinib-induced hepatitis and main strategy approaches. A complex score-based decision algorithm for choosing the further strategy approach after acute toxic imatinib-induced hepatitis in patients with CML has been presented. The following parameters should be assessed: the grade of hepatotoxicity reaction, the presence of liver transplantation or imatinib-induced liver cirrhosis and its possible pathogenetic mechanism, the presence of early molecular response (EMR) to imatinib therapy defined as three-month *BCR-ABL1* ≤10% according to the international scale (*BCR-ABL1^IS^*) or/and six-month *BCR-ABL1*^*IS* ^<1%; and the presence of the offender concomitant drug that probably caused the drug interaction with imatinib and the presence of viral hepatitis reactivation identified by polymerase chain reaction (PCR).

## Introduction and background

The targeted drugs called tyrosine kinase inhibitors (TKIs) such as imatinib, nilotinib, dasatinib, and others are currently the main treatment for chronic myeloid leukemia (CML). TKIs have been observed to promote significant improvements in the long-term survival rates since 2001. TKIs can promote achieving the deep molecular response in patients with CML. TKIs are a class of drugs that block the action of the mutant tyrosine kinase, a product of the *BCR-ABL1* fusion gene. Imatinib was the first drug from the TKIs group allowed for clinical use. Currently, imatinib is the gold standard of first-line therapy for Ph-positive CML patients over the world [1-2]. However, in rare cases, patients develop acute hepatitis and liver failure caused by imatinib therapy [3]. An increase in serum aminotransferase levels more than five times the upper limit of normal (ULN) has been registered only in 2% to 4% of patients treated for two to six months or more [1-4]. Nowadays, it is a great problem of gastroenterology, hepatology, and hematology due to the association with the high-level mortality and liver transplantations.

Every year, authors from different countries publish their experience of treatment of imatinib-induced hepatoxicity; to date, more than 25 clinical cases of imatinib-induced hepatitis have been published. Currently, there are conflicting data in the literature; in some cases, it was possible to resume the imatinib therapy, while in others, hepatotoxicity resumed after imatinib was restarted and it was necessary to replace imatinib with another TKI in a few cases [5-17]. In addition, follow-up of such patients after imatinib-induced hepatitis and the peculiarities of the second-line therapy have not been widely discussed in publications. There are a few publications about the experience of administration of second-line TKIs after acute hepatotoxicity, and in several reports, imatinib resumption without the recurrence of hepatotoxicity and with the recurrence of hepatotoxicity has been discussed. The majority of recommendations discuss only the imatinib failure approaches. In European LeukemiaNet recommendations for the management and avoidance of adverse events of treatment in CML in 2016, the general approaches to the management of adverse events (AEs) have been discussed [18]. However, it has still not been identified for what patients we can resume the imatinib therapy or choose the second-line therapy and what should be taken into account to make a decision for each person. A complex score-based decision algorithm for choosing further strategy approach after the acute toxic imatinib-induced hepatitis for patients with CML has been presented herein.

## Review

Guidelines for adverse event management in CML patients

Due to the meta-analysis of 12 published clinical trials of TKIs, hepatotoxicity manifested as low-grade increases in serum alanine (ALT) and/or aspartate transaminases (AST) in 25% to 35% of patients and as high-grade (grade three or four) increases in these transaminases in approximately 2% of patients [19]. Most of the patients with CML have manageable liver toxicity meaning that can be balanced by the dose interruption, dose reduction, and some symptomatic treatment, and it can be possible to keep most patients on treatment with TKI. The major, grade-three/four adverse effects typically occur during the first phase of the treatment, and in prevalence, cases are manageable but may require temporary treatment discontinuation and dose reduction, sometimes can lead to the treatment discontinuation in about 10% of patients [20].

Today, the choice of further therapy in CML patients after the acute hepatotoxicity is still a difficult question, which requires a complex individual approach based on the clinical guidelines of AE management. In addition, one of the questions is about the necessity and safety of the resumption imatinib therapy after the acute hepatotoxicity.

In 2016, general approaches to the management of AEs and avoidance of adverse events of the treatment in chronic myeloid leukemia were published in European LeukemiaNet recommendations [18]. According to this guidelines, grade-three AE requires withholding the TKI until the grade falls to less than three, and then resuming at the next lower level or withholding the drug until the severity falls to less than two, and resumes at the same dose. And if there is no resolution of AE within four weeks, the TKI should be stopped, and switched to another more appropriate one. In the case of the third episode of grade three, the guideline approach is the discontinuation and switching to another TKI. Grade-four AE requires the TKI stop and switch to another more appropriate TKI.

Published clinical cases follow-up after the acute imatinib hepatitis in CML patients

According to the Pub Med analyses, there are more than 25 clinical cases of the acute imatinib hepatitis, however, a small number of cases devoted to the monitoring after the acute imatinib hepatotoxicity in patients have been published. There are publications devoted to further therapeutic approaches and their outcomes, but only several are devoted to the switching to the second-line TKI and some to the imatinib therapy restarting [5-17].

For some patients, the imatinib therapy was restart without the recurrence of hepatotoxicity, while in some patients, the resumption imatinib therapy was accompanied by the recurring of the hepatotoxicity with the necessity of prednisolone therapy.

James C and co-authors reported two clinical cases of the acute imatinib hepatitis resolving with imatinib stopping and recurring in both with the restarting imatinib therapy [5].

Rocca P. and co-authors also reported the resolving acute imatinib hepatitis in three months after the imatinib discontinuation and recurring in two weeks after the restarting imatinib therapy [6].

Al Sobhi E and co-authors reported improving the liver function in a CML patient during the stopping of imatinib and worsening again during the restarting three weeks later, and then persisting for several months despite the stopping, responding ultimately to the prednisolone therapy [7].

However, Ikuta K and co-authors described the recurring hepatoxicity upon the restarting imatinib therapy at a low dose, but not recurring when prednisone (20 mg/day) was given and later the hormone therapy was tapered and stopped, then there was the resumption of the imatinib therapy with achieving the cytogenetic response without the development of hepatotoxicity [8].

Ferrero D and co-authors also described five cases of ALT and AST elevations in CML patients who had 25-40 mg corticosteroids per day. The hepatotoxicity was stopped after 3-8 weeks, that allowed the resumption of the full dose of imatinib than corticosteroids were gradually discontinued after 3-5 months without the recurrence of hepatotoxicity (Table 1) [9].

**Table 1:** Published сlinical cases follow-up after the resumption of imatinib therapy after acute imatinib hepatitis of CML patients CML, chronic myeloid leukemia

Authors, publication year	Clinical case	
The resumption of the imatinib therapy with the recurrence of hepatotoxicity		
James C. and co-authors (2003) [5]		Recurring imatinib hepatitis in both cases with the restarting imatinib therapy
Rocca P. and co-authors (2004) [6]		Imatinib hepatitis recurring in two weeks after the restarting imatinib therapy
Sobhi E. and co-authors (2007) [7]		Imatinib hepatitis worsening again during the restarting three weeks later resumption imatinib therapy
Ikuta K. and co-authors (2005) [8]		Recurring hepatoxicity upon the restarting imatinib therapy at a low dose
The resumption of the imatinib therapy without the recurrence of hepatotoxicity		
Ikuta K. and co-authors (2005) [8]		Hepatoxicity did not recur when prednisone (20 mg/day) was given. Later it was tapered and stopped, then there was the resumption of the imatinib therapy without the development of the hepatotoxicity
Ferrero D. and co-authors (2006) [9]		The resumption of the full dose imatinib while corticosteroids were gradually discontinued after three-five months without the recurrence of the hepatotoxicity (five clinical cases were described).
Kang B.W. and co-authors (2009) [10]		The acute imatinib hepatitis and hepatitis B reactivation required the liver transplantation. The patient was treated with lamivudine and seven months after the transplantation imatinib was restarted without further complications
Wang Y.D. and co-authors (2012) [11]		Symptomatic reactivation of hepatitis B in 40-year-old man six months after the starting of the imatinib therapy for CML responding to entecavir and able to continue imatinib
Lai G.-M. and co-authors (2013) [12]		Two cases of hepatitis B reactivation in CML patients receiving imatinib responded to the therapy with entecavir and the resumed therapy with the imatinib therapy

The resumption of the imatinib therapy started after resolving acute imatinib hepatotoxicity in described clinical cases.

The resumption of imatinib therapy requires great caution and prolonged careful monitoring of the patient. According to our opinion, it is not rational to restart imatinib therapy for the patients after the acute imatinib hepatotoxicity who did not have an early molecular response (EMR), defined as three-month *BCR-ABL1* ≤10% according to the international scale (*BCR-ABL1^IS^*) of treatment for the imatinib therapy. The importance and predictive value of the EMR have already been discovered in some clinical trials based on several years monitoring of patients [21]. The patients with EMR failure had lower rates of molecular response in comparison with patients who achieved EMR. The impact of EMR was reported by Marin et al. who analyzed the data from 282 with newly diagnosed CML chronic-phase patients treated with the first-line imatinib. *BCR-ABL1 *transcript level in three months strongly predicted overall survival, progression-free survival, and event-free survival, as well as correlating with the likelihood of achieving the complete cytogenetic response (CCyR), major molecular response (MMR), and complete molecular response (CMR) [22]. Marin and co-authors demonstrated that a *BCR-ABL1^IS^* transcript level more than 9.84% at three months was associated with higher probability of eight-year overall survival compared to those patients who achieved a *BCR-ABL1^IS^* transcript level below this cutoff (56.9% vs 93.3%, *P *< 0.001). Also, Hanfstein et al. demonstrated the importance of EMR based on follow-up of 1,303 patients on the first-line imatinib treatment as a part of the German CML IV study [23]. In that study, three different risk groups defined by *BCR-ABL1* transcript level in three months were analyzed: five-year overall survival (OS) rate was 87% in patients with a *BCR-ABL1^IS ^*>10%; 94% in patients with a *BCR-ABL1^IS^*1% to 10% (*P *= 0.012), and 97% in remaining patients with *BCR-ABL1^IS ^*<1%. However, there was no statistically significant difference between outcomes in the *BCR-ABL1^IS^*<1% group in comparison with the *BCR-ABL1^IS^* 1% to 10% group. Hence, the 10% cutoff was chosen as the most relevant landmark to denote a high-risk group of the patients [23].

The median development of the acute imatinib hepatotoxicity refers to a period of three to 12 months of therapy, which may be sufficient to assess the EMR the imatinib therapy and can serve as a risk factor stratification of each patient for the individual assessing the ratio risk/benefits imatinib therapy resumption after the acute imatinib hepatotoxicity.

The results of randomized clinical trials confirmed the importance of early switch of TKI based on EMR which can be useful as clinical strategies for the patient management especially after the acute hepatotoxicity more than five ULN in those patients for whom assessing risk/benefits plays a key role for outcomes [24].

More patients achieved EMR on nilotinib compared to imatinib, and after four years of follow-up, nilotinib continues to show higher rates of molecular response and lower rates of progression to accelerated phase or blastic phase compared to imatinib [21].

But for some patients, it is a necessity to switch on second-generation TKIs after the acute imatinib hepatotoxicity (Table 2). The role and safety of second-generation TKIs after the imatinib acute hepatotoxicity was discussed in some cases [16]. Nowadays, only in several described cases, after the imatinib hepatotoxicity, patients were treated with another TKI without the recurrence of liver injury [13-17].

**Table 2:** Published clinical cases follow-up after switching to second-line TKI after acute imatinib hepatitis in CML patients CML, chronic myeloid leukemia; TKI, tyrosine kinase inhibitor

Authors, publication year	Clinical case	Second-generation TKI
Perini G.F. and co-authors (2009) [13]	A 47-year-old CML woman with imatinib acute hepatotoxicity, which required successful liver transplantation	Treated with nilotinib without the recurrence of the liver injury
Spataro V. (2011) [14]	A 41-year-old woman with CML and postnecrotic imatinib-induced liver cirrhosis	Treated with nilotinib without the recurrence of the liver injury. the complete cytogenic response and major molecular response were achieved after one year
Harding D.J. and co-authors (2016) [15]	A 30-year-old CML woman with imatinib-induced fulminant liver failure which required successful liver transplantation	Treated with dasatinib at the standard 100 mg dose per day, then after two weeks, this dosage was decreased to 50 mg because of QTc interval prolongation associated with the dasatinib-induced inhibition of tacrolimus metabolism via the CYP3A4. Six months after the administration of dasatinib, the patient's BCR-ABL1 decreased to 0.37%, which was consistent with a major molecular response. Liver function tests remained normal.
Nacif L.S. and co-authors (2018) [16]	A 36-year woman with CML and the imatinib acute hepatotoxicity, which required liver transplantation	Treated with dasatinib due to the secondary myocardiopathy, 60 mg/day. No adverse effects were observed, and the patient’s BCR-ABL1 transcript reduced to 10.64% after two months
Lopina N. and co-authors (2018) [17]	A 56-year woman with CML and the imatinib acute hepatotoxicity	Treated with nilotinib in reduced dose (300 mg per day) without the recurrence of the liver injury. A major molecular response was achieved in six months.

Perini G.F and co-authors reported that after the imatinib acute hepatotoxicity in a 47-year-old woman with CML, who required liver transplantation the patient was treated with nilotinib without the recurrence of liver injury [13].

In 2011, Spataro V described the usage of nilotinib in a patient with postnecrotic liver cirrhosis related to imatinib. The patient received low-dose nilotinib (200 mg/d) and after two months, the nilotinib dosage was increased to 400 mg/d in two doses of 200 mg at 12-hour intervals. Seven months after the nilotinib administration the patient achieved the complete cytogenic response. Major molecular response was achieved after one year [14].

Harding DJ and co-authors described the successful use of dasatinib after the liver transplantation for imatinib-induced fulminant liver failure in CML patients. Dasatinib was implemented at the standard 100 mg dose per day, however two weeks this dosage was decreased to 50 mg because of QTc interval prolongation connected with the dasatinib-induced inhibition of tacrolimus metabolism via the CYP3A4. Six months after the implementation of dasatinib, the *BCR-ABL1^IS^* transcript level decreased to 0.37%, consistency with a major molecular response. Liver function tests remained normal [15].

In another clinical case, the patient was treated with dasatinib due to secondary myocardiopathy, 60 mg/day and any adverse effects were observed, and the patient’s *BCR-ABL1^IS^*transcript level reduced to 10.64% in two months [16].

The cross-intolerance of hepatotoxicity to other TKI is scarce. However, as mentioned above in patients treated previously with imatinib, with grades three-four or persistent grade two liver toxicity, the liver toxicity does not recurrent with the subsequent nilotinib therapy. In addition, nilotinib was used without the liver toxicity in a patient the undergoing liver transplantation for the fulminant liver failure associated with imatinib. Another TKI - dasatinib was also safely used after the patient had received imatinib and developing liver toxicity was successfully treated with glucocorticoids.

**Figure 1:**
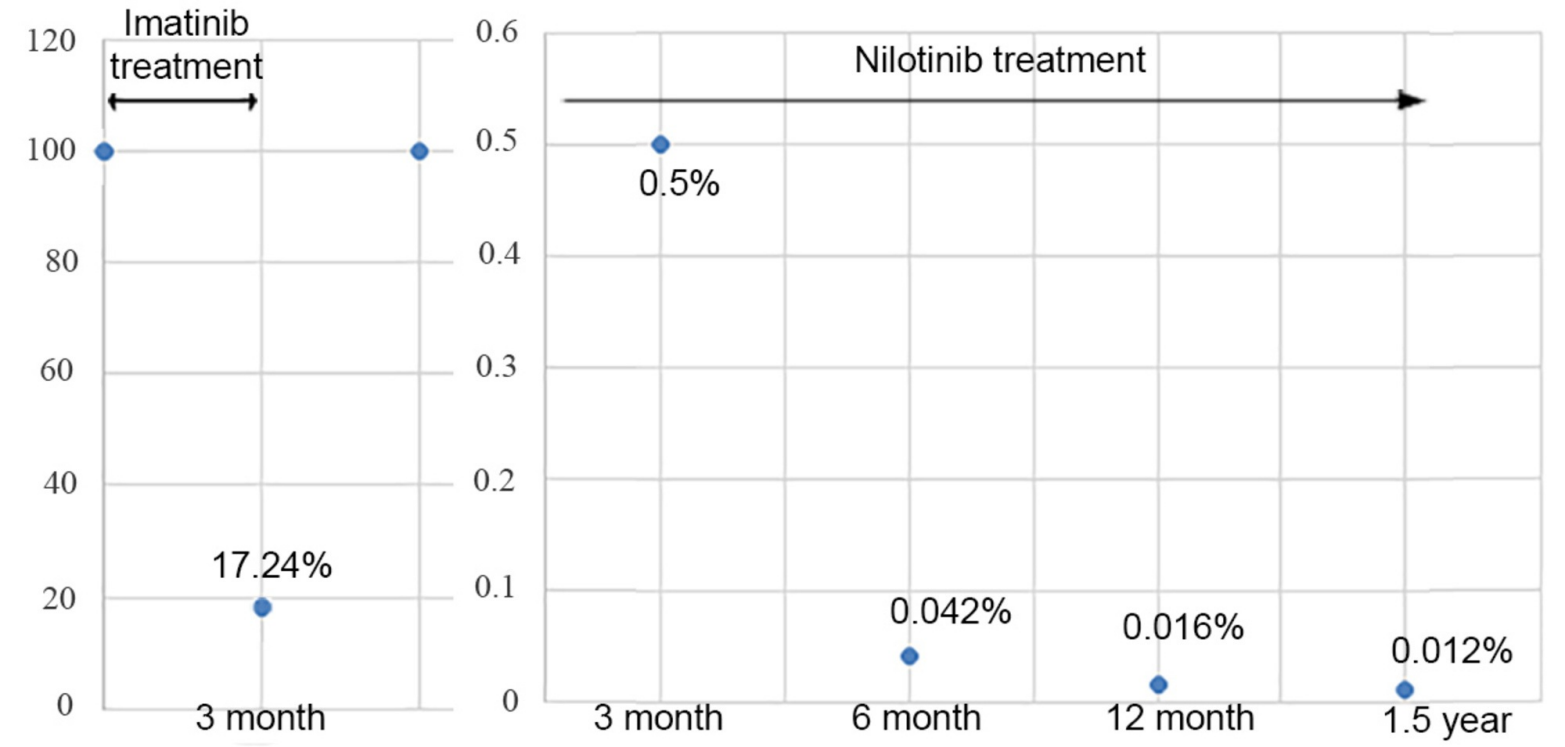
The BCR-ABL1IS transcript level in peripheral blood during one year and a half follow-up period in a patient with CML CML, chronic myeloid leukemia

Previously we described a clinical case of the acute toxic imatinib-induced hepatitis in a 56-year-old woman with chronic myeloid leukemia, concomitant sulfa allergy and rheumatoid arthritis occurred in 2017. Hepatotoxicity appeared after three months of imatinib treatment and three days after the increasing of the imatinib dosage from 400 mg per day to 600 mg per day, which is resolved within three months after the imatinib discontinuation and prednisolone administration [17]. In addition, we provided a two-year follow-up in this clinical case. After the resolving acute hepatitis in four months after the imatinib hepatoxicity started, nilotinib was prescribed in a reduced dosage -300 mg per day. After three months of the nilotinib treatment, the *BCR-ABL1^IS^* transcript level was 0.500% (the complete CCyR was achieved). After six months of the nilotinib treatment, the *BCR-ABL1^IS^* transcript level was 0.042% (the MMR was achieved). After one year of nilotinib treatment, the *BCR-ABL1^IS^* transcript level was 0.016%, after one year and a half the *BCR-ABL1^IS^* transcript level was 0.012% (Figure 1).

During a two-year follow-up, the nilotinib treatment in a reduced dosage 300 mg per day hepatotoxicity did not recur, however, the fasting blood sugar increase to 6.2-6.7 mmol/l was observed. Glycosylated hemoglobin was registered within normal levels of less than 5.9%.

The prevalence cases in which there was a switching TKI to the second-line drug were registered among patients after the successful liver transplantation or imatinib-induced liver cirrhosis.

The acute imatinib hepatotoxicity should be clearly stratified into possible mechanisms of its development, since it may be decisive in the choice of further tactics. Great attention should be paid to drug interactions and viral hepatitis reactivation.

In several reported cases of imatinib hepatotoxicity, liver injury was caused by the drug interactions, these patients can continue the imatinib therapy after the resolving hepatitis without offender concomitant drug usage, however, this interaction can be fatal [25-27].

Imatinib is metabolized in the liver and is facilitated by several isozymes of the cytochrome P450 system, including CYP3A4, CYP3A5 and, to a lesser extent, CYP1A2, CYP2D6, CYP2C9, and CYP2C19. CYP3A4 is the main enzyme for imatinib metabolism. CYPs 1A2, 2D6, 2C9 and 2C19 play a secondary role in its metabolism. Interactions between imatinib and inducers or inhibitors of these enzymes may occur, which can lead to changes in the plasma concentration of imatinib and co-administered drugs. According to the peculiarities of the imatinib metabolism which is based on isoenzyme CYP3A4 of the cytochrome P450, while the imatinib prescribing it is necessary to take into account the inter-drug interactions and the metabolic features of other medications to exclude the potentiation or the attenuation of the imatinib action [28].

An increase in the imatinib concentration in plasma may occur when it’s simultaneously used with drugs that inhibit the cytochrome P450 isoenzyme CYP3A4. In healthy volunteers, there were an increase in Cmax by 26% and AUC by 40% when it was simultaneous the application of imatinib and ketoconazole, which is an inhibitor of CYP3A4 [28]. In contrast, the simultaneous use of drugs that are inducers of CYP3A4 (eg, dexamethasone), can lead an increase in the metabolism of imatinib and a decrease in its concentration in the plasma. During the simultaneous use of imatinib and simvastatin, the increase in Cmax and AUC of simvastatin in two and three point five is observed, respectively, which is a consequence of the inhibition of CYP3A4 by imatinib [28]. Also inducers of cytochrome P450 isoenzyme CYP3A4, such as phenytoin, can induce inadequate responses due to increased imatinib clearance and low imatinib trough plasma levels. Therefore, this interaction should be avoided. When it is not possible, it is necessary to increase the imatinib dose and to measure the plasma levels recommended, if it is available [28].

In vitro studies, it has also been shown that imatinib inhibits the isoenzyme CYP2D6 of cytochrome P450 at the same concentrations in which it inhibits CYP3A4. That’s why the possibility of amplification effect of drugs that are substrates of the isoenzyme CYP2D6 should be considered when they are used together with imatinib. Although no special studies have been conducted, nevertheless, great caution is recommended [28].

The drugs that may require dosage adjustment or special monitoring during the therapy include: antibiotics like clarithromycin, erythromycin or troleandomycin, rifampicin/rifabutin; prednisolone and dexamethasone; antifungals like itraconazole or ketoconazole; anticonvulsants like phenytoin, carbamazepine, clonazepam, or phenobarbital; antihypertensives like nifedipine, amlodipine, felodipine, isradipine, nimodipine; anti-anxiety agents like alprazolam, diazepam, or triazolam; cholesterol-lowering drugs like lovastatin, atorvastatin, or simvastatin and cyclosporin, pimozide, warfarin etc. [28]. Several cases of the developing acute toxic hepatitis, an acute hepatic failure and the lethal outcome in concomitant imatinib and acetaminophen usage are described [25,26].

James C. and co-authors reported a clinical case of marked ALT elevations (2430 U/L) in a 58-year-old woman treated with imatinib for CML for five months. The hepatitis was resolved and recurring with restarting the imatinib therapy [5]. Hepatotoxicity in this case according to the authors’ opinion could be caused by the drug interaction of imatinib and roxithromycin. It has been shown that roxithromycin is an inhibitor of the cytochrome P450 enzyme CYP3A4, although it is not as effective as erythromycin. Since imatinib is a competitive inhibitor of cytochrome P450 enzymes CYP3A4/5, CYP2C9, and CYP2D6, if it is combined with roxithromycin, the level of imatinib, thereby increasing its toxicity increases too. The patients experienced a recurrence of liver damage and an increase in serum transaminase levels after repeated of imatinib therapy.

Osorio S. et al. reported a case of the severe liver toxicity in a 63-year-old woman with CML probably induced by a drug interaction between imatinib and sertraline. The patient started treatment with sertraline three months after starting imatinib. From the beginning of the sertraline treatment, the patient had vomiting, and five weeks later a severe hepatic failure (AST 782 IU/l, ALT 880 IU/l, Alk P 457 IU/l, total bilirubin 10 mg/dl) developed which was resolved after the treatment with prednisone and ursodeoxycholic acid, the chronic liver disease stage IV (cirrhosis) without signs of portal hypertension developed [27].

Also, several cases of reactivation of chronic hepatitis B due to the imatinib treatment in patients with inactive hepatitis B or with HBsAg carriers prior to the initiation of imatinib therapy have been described [10-12,29-32]. Clinical manifestations, as a rule, are accompanied by the development of the acute cytolysis syndrome with a marked increase in serum ALT and minimal changes in the level of alkaline phosphatase. Typically, hepatitis B virus (HBV) DNA is present in increasing amounts in the serum as compared to the latent phase in the early stage of reactivation, which rapidly decreases to its former level with the recovery. Positive antibodies of the IgM class (IgM anti-HBc) can also be detected in the blood during the inactivation hepatitis B. However, the reactivation of the hepatitis B caused by the imatinib therapy may also be accompanied by the development of the severe acute hepatitis with a fatal outcome.

The reactivation of hepatitis B is probably caused by suppression of immunity, that leads to the enhanced replication of the virus, followed by restoration of immunity and the development of the acute liver injury. It was known that the reactivation of HBV usually occurs due to the cancer chemotherapy or implementation of high doses of corticosteroids or rituximab. The reactivation can also be caused by antagonists of tumor necrosis factor, such as infliximab. The occurrence of hepatitis B reactivation during the imatinib therapy was a surprise, as this therapy is not considered to be a significant cause of immunosuppression and changes in the appearance of HBV replication were not expected. The reactivation of HBV with the imatinib therapy has been reported in patients who are HBsAg-positive, but initially have minimal manifestations of the chronic liver disease and low levels of HBV DNA, negative HBeAg in the serum.

Immunosuppression is accompanied by an increase in HBV replication rate with an increase in the amount of HBV DNA copies, HBeAg can also be detected again in serum. Subsequent partial restoration of the immunity is accompanied by an immunological response to HBV antigens. Currently, several very convincing clinical cases of the hepatitis B reactivation associated with the imatinib therapy that occurred after three months and more than a year after the initiation of the imatinib therapy have been described. the Reactivation of hepatitis B can be severe and accompanied by at least a 10% mortality rate among such patients [10-12,29-32].

There are several reported clinical cases devoted to the reactivation of hepatitis B due to the imatinib therapy in which the imatinib therapy was restarted after the resolving acute liver injury, even after the liver transplantation.

Kang B.W. and co-authors described the severe reactivation of hepatitis B due to imatinib therapy in a 48-year-old man with CML and chronic hepatitis B, which manifested in the acute liver failure, that required the liver transplantation. Despite the stopping imatinib and starting lamivudine, his condition deteriorated with a steady rise of serum bilirubin to a peak of 53 mg/dL, worsening prothrombin time and appearance of the hepatic encephalopathy. A living donor liver transplantation was done approximately two months after the presentation with jaundice. In follow up, his serum bilirubin and enzymes remained normal and HBV DNA fell to the undetectable levels. He remained on lamivudine and in seven months was restarted on imatinib without further complications [10].

Wang YD and co-authors described the symptomatic reactivation of hepatitis B in a 40-year-old man six months after the starting of the imatinib therapy for CML (bilirubin 3.0 mg/dL, ALT 1011 U/L, INR normal, HBV DNA 285,000 IU/mL), responding to entecavir and it was possible to continue imatinib [11].

Lai G.-M. et al. reported two cases of the hepatitis B reactivation in the CML patients receiving imatinib responded to the therapy with entecavir and resumed the imatinib therapy [12].

Factors for the assessment after the acute toxic imatinib-induced liver injury in the chronic myeloid leukemia patients in decision-making approach

In our opinion, the choice of strategy approach after the firstly appeared acute imatinib hepatitis and necessity of the second-generation tyrosine kinase inhibitors should be personalized and based on several consecutive steps because a doctor should answer two main questions to make a decision:

1. Imatinib should be withdrawn or could be restarted?

2. What TKI should be chosen if the decision is to switch the therapy?

To answer the first question it is a necessity to take into account the following factors:

1. The grade of hepatotoxicity reaction and the presence of the liver transplantation or imatinib-induced liver cirrhosis and it’s a possible pathogenetic mechanism.

2. The presence of EMR to the imatinib therapy defined as three-month *BCR-ABL1^IS^ *≤10%;

3. The presence of EMR to the imatinib therapy on six-month defined as six-month *BCR-ABL1^IS^* < 1%;

4. The presence of the offender concomitant drug probably caused the drug interaction with further development of the hepatotoxicity;

5. The presence of the viral hepatitis reactivation identified by PCR (PCR+);

The first step decision approach after the acute toxic imatinib-induced liver injury in the chronic myeloid leukemia patients

If we add «Yes» or «No» to the mentioned factors assessment we’ll receive a personalized decision approach, that can facilitate daily activities of doctors (Table 3).

**Table 3:** The personalized decision approach in chronic myeloid leukemia patients after the acute toxic imatinib-induced liver injury after three months of imatinib treatment * This decision approach can be used in patients with after three months of imatinib treatment and after the firstly appeared acute imatinib hepatitis or I-II grade of imatinib hepatotoxicity recurrence, if imatinib hepatitis recurs in III-IV grade of hepatotoxicity the imatinib withdrawal should be considered; Patients with the presence of the liver transplantation or imatinib-induced liver cirrhosis caused by autoimmune mechanism only cannot be evaluated by the algorithm. All decisions to restart should be made after the resolving acute hepatitis and after the transaminases normalization as in previously reported cases; ** Imatinib can be restarted in the reduced or the same dose; *** «Yes» – when hepatoxicity stops less than in one month and does not recurs, «No» - when the hepatoxicity does not stop after one month or recurs; **** Not applicable if the hepatotoxicity developed earlier than in six months. EMR, early molecular response

Factors	Imatinib restart*	Imatinib withdrawal
The grade of hepatotoxicity reaction		
I grade	Yes	No
II grade	Yes**	No
III grade	Yes/No***	Yes/No
IV grade / the presence of the liver transplantation or imatinib-induced liver cirrhosis caused by drug interaction or viral hepatitis reactivation	No	Yes
The presence of EMR to the imatinib therapy defined as three-month BCR-ABL1^IS^ ≤10%:		
Yes	Yes	No
No	No	Yes
The presence of EMR to the imatinib therapy defined as six-month BCR-ABL1^IS^ <1% (if applicable****):		
Yes	Yes	No
No	No	Yes
The presence of the offender concomitant drug probably caused drug interaction		
Yes	Yes	No
No	No	Yes
The presence of the viral hepatitis reactivation identified by polymerase chain reaction (PCR+)		
Yes	Yes	No
No	No	Yes

If we replace Yes/No values with one and zero we can receive the score decision algorithm for patients after three months of imatinib treatment (Table 4).

**Table 4:** The score-based decision approach in the chronic myeloid leukemia patients after the acute toxic imatinib-induced liver injury after three months of imatinib treatment (points) * This decision approach can be used in patients with after three months of imatinib treatment and after the firstly appeared acute imatinib hepatitis or I-II grade of imatinib hepatotoxicity recurrence, if imatinib hepatitis recurs in III-IV grade of hepatotoxicity the imatinib withdrawal should be considered; Patients with the presence of the liver transplantation or imatinib-induced liver cirrhosis caused by autoimmune mechanism only cannot be evaluated by the algorithm. All decisions to restart should be made after the resolving acute hepatitis and after the transaminases normalization as in previously reported cases; ** Imatinib can be restarted in the reduced or the same dose; *** «Yes» – when hepatoxicity stops less than in one month and does not recurs, «No» – when the hepatoxicity does not stop after one month or recurs; **** Not applicable if the hepatotoxicity developed earlier than in six months.

Factors	Imatinib restart*	Imatinib withdrawal
The grade of hepatotoxicity reaction		
I grade	1	0
II grade	1**	0
III grade	1/0***	1/0
IV grade / the presence of the liver transplantation or imatinib-induced liver cirrhosis caused by drug interaction or viral hepatitis reactivation	0	1
The presence of EMR to the imatinib therapy defined as three-month BCR-ABL1^IS^ ≤10%:		
Yes	1	0
No	0	1
The presence of EMR to the imatinib therapy defined as six-month BCR-ABL1^IS^ <1% (if applicable****):		
Yes	1	0
No	0	1
The presence of the offender concomitant drug probably caused drug interaction		
Yes	1	0
No	0	1
The presence of the viral hepatitis reactivation identified by polymerase chain reaction (PCR+)		
Yes	1	0
No	0	1

The following parameters should be assessed: the grade of hepatotoxicity reaction (I - one point, II - one point, III - one/zero points (one point - when hepatoxicity stops less than in one month, zero points - when the hepatoxicity does not stop after one month), IV/the presence of the liver transplantation or imatinib induced liver cirrhosis caused by drug interaction or viral hepatitis reactivation - zero points); the presence of early molecular response to the imatinib therapy defined as three-month *BCR-ABL1^IS^*≤10% (if Yes - one point, if No - zero points); the presence of EMR to the imatinib therapy defined as six-month *BCR-ABL1^IS^*^ ^<1% (if applicable because the imatinib injury can occur earlier than in six months) - if Yes- one point, if No - zero points; the presence of the offender concomitant the drug probably caused drug interaction with imatinib (if Yes- one point, if No - zero points); the presence of the viral hepatitis reactivation identified by PCR (PCR+) - if Yes- one point, if No - zero points.

If the total patient’s score due to imatinib restart arm is zero points - imatinib should be withdrawn and tyrosine-kinase inhibitor should be switched; one point - the imatinib withdrawal should be considered (if imatinib treatment continues more than six months withdrawn preferred, if less than six months can be restart); if total points ≥2, imatinib can be restarted after the acute toxic imatinib-induced liver injury resolving.

Imatinib restarting without hepatotoxicity recurrence observed in previously described clinical cases with tapered glucocorticosteroids. The simultaneous glucocorticosteroids usage can be considered in a patient with the decision to restart imatinib and the imatinib hepatotoxicity was treated with glucocorticosteroids.

Due to the date from PubMed and LiverTox, imatinib hepatotoxicity clinical cases occur in the patients treated with imatinib less than three months in 24%. In these situations, we could not base on the EMR, but only on the grade of hepatotoxicity and possible pathogenic mechanism the Modified Score-based Decision Approach could be proposed (Table 5).

**Table 5:** The modified score-based decision approach in the chronic myeloid leukemia patients after the acute toxic imatinib-induced liver injury treated with imatinib less than three months (points) * This decision approach can be used in patients with less than three month of imatinib treatment and after the firstly appeared acute imatinib hepatitis or I-II grade of imatinib hepatotoxicity recurrence, if imatinib hepatitis recurs in III-IV grade of hepatotoxicity the imatinib withdrawal should be considered; All decisions to restart should be made after the resolving acute hepatitis and after the transaminases normalization as in previously reported cases; ** Imatinib can be restarted in the reduced or the same dose; *** «Yes» – when hepatoxicity stops less than in one month and does not recurs, «No» – when the hepatoxicity does not stop after one month or recurs.

Factors	Imatinib restart*	Imatinib withdrawal
The grade of hepatotoxicity reaction		
I grade	1	0
II grade	1**	0
III grade	1/0***	1/0
IV grade	0	1
The presence of the offender concomitant drug probably caused drug interaction		
Yes	1	0
No	0	1
The presence of the viral hepatitis reactivation identified by Polymerase Chain Reaction (PCR+)		
Yes	1	0
No	0	1

If the total patient’s score due to imatinib restart arm is zero points - imatinib should be withdrawn and tyrosine-kinase inhibitor should be switched; one point and more - imatinib can be restarted after the acute toxic imatinib-induced liver injury resolving.

In previously reported clinical case the patient had the absence of the early molecular response after three months of the imatinib therapy - the *BCR-ABL1^IS^*transcript level in peripheral blood was 17.241% [20]. Based on the presented algorithm if we calculate our previously reported clinical case we would receive zero point total score due to imatinib restart arm, which would confirm the imatinib withdrawal and tyrosine-kinase inhibitor switch. So, based on the absence of the early molecular response, the grade of hepatotoxicity and other factors our clinical team decided to choose the second-generation tyrosine kinase inhibitor (nilotinib) right after the improving liver tests (Table 6).

**Table 6:** The example of a score based decision approach in a chronic myeloid leukemia patient after the acute toxic imatinib-induced liver injury (imatinib withdrawal) N/A- not applicable

Factors	Imatinib restart*
The grade of hepatotoxicity reaction	
I grade	
II grade	
III grade	
IV grade / the presence of the liver transplantation or imatinib-induced liver cirrhosis caused by drug interaction or viral hepatitis reactivation	0
The presence of EMR to the imatinib therapy defined as three-month BCR-ABL1^IS^ ≤10%:	
Yes	
No	0
The presence of EMR to the imatinib therapy defined as six-month BCR-ABL1^IS^ <1% (if applicable****):	N/A
Yes	
No	
The presence of the offender concomitant drug probably caused drug interaction	
Yes	
No	0
The presence of the viral hepatitis reactivation identified by Polymerase Chain Reaction (PCR+)	
Yes	
No	0

At the end of 2018, we observed another case of the acute imatinib hepatotoxicity in a 32-year-old woman with CML. The acute imatinib hepatotoxicity grade III (ALT 354 ul/l, AST 183 ul/l) developed after six months of the imatinib treatment. After three months of the imatinib treatment, the* BCR-ABL1^IS^*transcript level in peripheral blood was 0.514% (the complete CCyR was achieved). The *BCR-ABL1^IS^* transcript level in peripheral blood was 0.107 % after six months of the imatinib treatment (the MMR was achieved). So, EMR was registered after three and six months of the imatinib treatment. The hepatotoxicity grade III can be resolved within two weeks imatinib withdrawal (less than one month) on 20 mg prednisolone that was gradually tapered and withdrawal after 45 days of the hormone treatment. Viral hepatitis by PCR was negative, any offender concomitant drug probably caused the drug interaction was identified. The imatinib-induced liver cirrhosis was not identified. Imatinib was restarted in a reduced dose of 300 mg per day after the resolving hepatitis with the simultaneous prednisolone treatment that was gradually tapered and withdrawn. During four months of the imatinib restarting liver tests monitoring was within normal ranges. Imatinib dosage was increased to 400 mg per day after four months of the imatinib restarting. The example of this case approach score was calculated as three points, so the imatinib treatment was restarted (Table 7).

**Table 7:** The example of the score based decision approach in a chronic myeloid leukemia patient after the acute toxic imatinib-induced liver injury (imatinib restart)

Factors	Imatinib restart*
The grade of hepatotoxicity reaction	
I grade	
II grade	1
III grade	
IV grade / the presence of the liver transplantation or imatinib-induced liver cirrhosis caused by drug interaction or viral hepatitis reactivation	
The presence of EMR to the imatinib therapy defined as three-month BCR-ABL1^IS^ ≤10%:	
Yes	1
No	
The presence of EMR to the imatinib therapy defined as six-month BCR-ABL1^IS^ <1% (if applicable****):	
Yes	1
No	
The presence of the offender concomitant drug probably caused drug interaction	
Yes	
No	0
The presence of the viral hepatitis reactivation identified by Polymerase Chain Reaction (PCR+)	
Yes	
No	0

Due to general approaches to the management of AEs and avoidance of the adverse events of the treatment in chronic myeloid leukemia in the case of grade three hepatotoxicity if the decision is to restart imatinib it can be used in a reduced dosage, the two grade of hepatotoxicity if imatinib was restarted it can be used in the same prior dosage [18].

The second step decision approach after the acute toxic imatinib-induced liver injury in the chronic myeloid leukemia patients if the decision is to switch the therapy.

The second question is «What TKI should be chosen?» if the decision is to switch the therapy?

To answer the second question, it is a necessity to take into account the following factors:

1. Concomitant diseases and conditions;

2. The mutations of the* BCR-ABL1 *kinase domain (KD) (if it is indicated).

The presence of the following comorbidities and conditions should be assessed (Table 8):

**Table 8:** Comorbidities and conditions for the assessment to choose the second-line TKI 0 – should avoid, 1 point – preferred, 0.5 – intermediate, ND – no data TKI, tyrosine kinase inhibitor

Condition Drug	Nilotinib	Dasatinib	Ponatinib	Bosutinib
Coronary artery disease (documented atherosclerotic coronary artery lesion or myocardial infarction in anamnesis) or cerebrovascular disease	0	0	0	1
Peripheral arterial occlusive disease	0.5	1	0	1
A prior venous thrombosis	1	1	0	1
A high/very high cardiovascular risk	0	1	0	1
A heart failure (proBNP, left ventricular dysfunction)	1	1	0	1
Cardiac rhythm alterations (QT prolongation syndromes, Torsades de pointes documented prior, concomitant drugs that prolong QT)	0	1	1	1
An uncontrolled hypertension	1	1	0	1
Pulmonary pleural effusion, pulmonary (lung) disease disorders (e.g. chronic obstructive pulmonary disease)	1	0	1	0.5
Pneumonitis	1	0	1	0
Pre-existing pulmonary hypertension	1	0	1	1
A prior hepatotoxicity	1	1	0	1
A prior hyperbilirubinemia Genotypic (UDP- glucuronyltransferase) – Gilbert syndrome	0	1	1	1
T2DM controlled or prediabetes	1	1	1	1
T2DM uncontrolled	0	1	1	1
Family hypercholesterinemia or high baseline total cholesterol	0	1	0 (TG increase)	1
Hypophosphatemia, hyperparathyroidism	0	0.5	1	1
Hypothyroidism	0	0	1	1
Hematological myelosuppression				
Anemia	1	0	0.5	1
Thrombocytopenia	0.5	0	0	0.5
Neutropenia	0.5	0	0.5	0.5
Sepsis, infections complications	1	0	1	1
The bleeding history, gastrointestinal bleeding	1	0	0	1
The Anticoagulation concomitant necessity ( concomitant AF)	1	0	0	1
The antiplatelet therapy necessity	1	0	0	1
Diarrhea	0.5	1	1	0
Pancreatic problems (history of pancreatitis)	0	1	0	1
A reproductive function	1	0	ND	0
A renal failure	1	0.5	0	0
Neurological conditions (headache)	0	0	0	0.5

· Coronary artery disease (documented atherosclerotic coronary artery lesion or myocardial infarction in anamnesis) or cerebrovascular disease)

· The cardiovascular risk assessment, indicating the high/very high patients (asses fasting glucose, HbA1c, lipids (cholesterol, low- and high-density lipoprotein (LDL and HDL) and triglycerides), creatinine), and the repetition of these parameters every six/12 months when a therapeutic regimen including ponatinib or nilotinib is chosen;

· Peripheral arterial occlusive disease (claudication, assessment based on Rutherford classification, ankle-brachial index assessment or duplex ultrasonography to asses asymptomatic peripheral arterial occlusive disease);

· The prior venous thrombosis;

· High/very high cardiovascular risk ;

· Cardiac function (cardiomyopathy): proBNP, left ventricular dysfunction;

· Cardiac rhythm alterations (assessment of QT interval, assessment of potassium and magnesium serum concentrations, QT prolongation syndromes, Torsade de pointes prior documented, concomitant drugs that prolong QT), great caution when they are used with CYP3A4 inhibitors;

· Uncontrolled hypertension;

· Pulmonary pleural effusion, pulmonary (lung) disease disorders (e.g. chronic obstructive pulmonary disease), pre-existing pulmonary hypertension;

· Hepatobiliary - prior hepatotoxicity, the present condition of liver function, prior hyperbilirubinemia, genotypic (UDP- glucuronyltransferase) - Gilbert syndrome;

· Endocrine conditions and diseases - glycemic status, type two diabetes mellitus (T2DM) controlled or uncontrolled, hypophosphatemia, hyperparathyroidism, hypothyroidism;

· Baseline hematological myelosuppression;

· Sepsis, infections complications;

· Bleeding history, gastrointestinal bleeding;

· Anticoagulation concomitant necessity (concomitant AF);

· The necessity of antiplatelet therapy;

· Diarrhea;

· The primary assessing of the pancreas state, the history of pancreatitis;

· Reproductive function;

· Renal failure.

If the condition or disease is present, all the second-line drugs are assessed. The general score is the sum of concomitant conditions and diseases scores.

The drug gains the more points, the more it will be preferable to use in a patient.

 According to the European LeukemiaNet Recommendations 2013, the use of mutation analysis of BCR-ABL1 KD is indicated in the following situations [20]:

· During the first-line therapy, an analysis should be done in the case of failure and in the case of an increase in the BCR-ABL1 transcript level leading to the loss of MMR.

· During the second-line therapy, the analysis should be done in case of the hematological or cytogenetic relapse or in case of pre-existing mutations.

· In case of accelerated phase or blastic phase, it always should be done.

Resistance and sensitivity of TKI drugs based on a *BCR-ABL1 KD *mutation profile in the CML patients are showed in Table 9 [33].

**Table 9:** The resistance and sensitivity of TKI drugs based on a mutation profile in the chronic myeloid leukemia patients «-» resistance, «+» - sensitivity TKI, tyrosine kinase inhibitor

Drug Mutation profile	Nilotinib	Dasatinib	Ponatinib	Bosutinib
T315I	-	-	+	-
T315A	+	-	+	+
Y253H E255K/V F359C/V/C/I	-	+	+	+
V299L	+	-	+	-
F317L/V/I/C	+	-	+	+

The limitations of our non-systematic revision and proposed algorithm are the absence of external clinical validation and scarce date previous reported imatinib hepatotoxicity cases. Currently, there are no possibilities to provide validation based on the published clinical cases of imatinib hepatotoxicity due to the absence in them the three- and six-month EMR date. Further clinical validation is necessary based on the proposed algorithm. In addition, it would be necessary to create a world register of imatinib hepatotoxicity, where all data on the possible mechanism, dosage, grade of hepatotoxicity, presence of EML, etc. would be presented. The world register of imatinib hepatotoxicity can be helpful in clinical validation of the proposed algorithm because there are more than 25 publications of imatinib hepatotoxicity at this date and it is more and more difficult to publish clinical case articles devoted imatinib hepatotoxicity, but further patient’s management strategy approach does not exactly clear.

## Conclusions

Currently, five TKIs have been approved for clinical use in CML patients, which include imatinib, nilotinib, dasatinib, ponatinib, and bosutinib. The choice of further CML therapy in patients after acute hepatotoxicity is still a difficult question, which requires a complex individual approach based on the clinical guidelines for adverse event management. Data about the further follow-up strategy approach in the CML patients after the acute toxic imatinib-induced liver injury are concerning and at times controversial. We discussed all the available data devoted to the resumption of imatinib therapy after acute hepatotoxicity with and without the recurrence of hepatotoxicity and switching to another TKI. There are no clear data on which factors the physician’s decision should be based on CML patients after the acute toxic imatinib-induced liver injury. Imatinib should be restarted or withdrawn, when and for whom the second-line therapy should be started. The physician’s decision is usually based on the published data of similar cases, personal experience, and severity of hepatotoxicity. First, we describe a complex score-based decision algorithm for choosing the further strategy approach after the acute toxic imatinib-induced hepatitis in patients with CML firstly present. The following parameters should be assessed: the grade of hepatotoxicity reaction, the presence of liver transplantation or imatinib-induced liver cirrhosis and its possible pathogenetic mechanism, the presence of early molecular response to imatinib therapy defined as three-month *BCR-ABL1^IS ^*≤10%, the presence of EMR to imatinib therapy defined as six-month *BCR-ABL1^IS^<*1%, and the presence of the offender concomitant drug probably causing the drug interaction with imatinib and the presence of the viral hepatitis reactivation identified by PCR.

The second- and third-generation TKIs have not been compared head-to-head. The selected one of the other should be based on the concomitant diseases and conditions, side-effect profiles, *BCR-ABL1 KD*mutation profiles, and drug interactions. The presented novel score-based decision approach in the CML patients after the acute toxic imatinib-induced liver injury can facilitate the daily activities of doctors in the CML patients after the acute liver injury.
